# Retraction: Prevalence of pulmonary hypertension and its associated factors among chronic obstructive pulmonary diseases patients at public hospitals of Addis Ababa, Ethiopia, 2024: a facility-based cross-sectional study

**DOI:** 10.3389/fpubh.2025.1652830

**Published:** 2025-06-30

**Authors:** Frontiers Editorial Office

The journal retracts the November 26 2024 article cited above.

Following publication, it was brought to our attention that the authors used and published primary research data, which was generated by other researchers, without permission and without a mention to their contributors. Unauthorized data use is a breach of Frontiers' guidelines and those of the Committee on Publication Ethics. As such, this article is being retracted.

The authors agree to this retraction.

Frontiers would like to thank Hailemichael Kindle Abate leading the study presented for contacting the journal regarding the misappropriation of the data in the published article.

